# Dental complications in homocystinurias

**DOI:** 10.1016/j.ymgmr.2023.100999

**Published:** 2023-08-14

**Authors:** Kimberly A. Chapman, Danae Bartke, Vanessa Vogel-Farley, Mary Cobb, Mary Chapman

**Affiliations:** a Children's National Rare Disease Institute, Washington, DC, United States of America; b HCU Network America; c Global Genes: RARE-X Program; d UPMC Children's Hospital of Pittsburgh, Department of Pediatric Dentistry, Pittsburgh, PA, United States of America

**Keywords:** Homocystinuria, Dental, Oral health, Cystathionine beta synthase deficiency, Methylene tetrahydrofolate reductase deficiency, Cobalamin processing defects

## Abstract

**Background:**

Cystathionine beta synthase deficiency (causing classical homocystinuria) has been associated with high-arched palates and crowded teeth, but little has been said about other oral health complications. Other homocystinurias (*e.g.*, the remethylation defects) also have had little reported in terms of oral health. Individuals with the homocystinurias have been described as having bone density issues which can correlate with oral health. Moreover, elevations in homocysteine have a theoretical impact on tooth health and the paucity of clinical reports of oral health issues in homocystinuria may be the consequence of lack of attention by the medical community.

**Significance:**

Oral health is essential to overall health. If inadequate attention is paid to the oral health complications which can be seen in homocystinurias, then appropriate referrals and attention in therapeutic guidelines will not reflect the importance of oral health.

**Specific aims/research question:**

What oral health complications are reported by individuals with homocystinurias? Do these differ according to diagnosis?

**Methods:**

Data were collected from patients with homocystinurias by a series of questionnaires using the RARE-X platform. All subjects were consented prior to the collection of their data. All research was performed in accordance with the Declaration of Helsinki. Demographic data were collected as the initial questionnaire and other data were collected *via* the oral health questionnaire.

**Analysis:**

Questionnaires were opened to the community in mid-2022 and collection of data for this study ended with data submitted up to November 2022. Descriptive statistics were done. Due to the small size of the cohort, additional statistical analyses were not attempted.

**Results:**

Patients with homocystinuria, not related to cystathionine beta synthase deficiency, are reporting some tooth structure differences. The cohort taken as a whole does not have increased risk for gingivitis, but there appears to be a risk for long-term gum disease possibly due to the rate of osteoporosis/osteopenia in this population. A large number of individuals have malalignment and malocclusion of the teeth. These data highlight oral health as an important component of care in individuals with the homocystinurias as is true of the general population at large.

## 1 Introduction

**Fig. 1:**
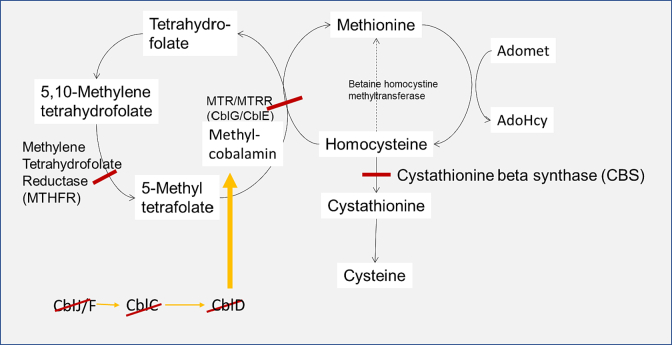
Cartoon illustrating the Homocystinurias including MTR/MTRR (also known as Cobalamin G and E), Methylene tetrahydrofolate reductase (MTHFR), and Cystathionine beta synthase (CBS) deficiencies as designated by the red lines. Cobalamin C, D, J and F deficiencies are also marked by red lines. Tetrahydrofolate is a folate derivative which is active in this pathway.

The metabolic homocystinurias include several dysfunctional enzymes within the methionine cycle including Cystathionine beta synthase deficiency (CBS deficiency, OMIM 236200), severe methylene tetrahydrofolate reductase deficiency (MTHFR, OMIM #236250); Cobalamin G deficiency also known as methionine synthase deficiency (MTR, OMIM #250940), and Cobalamin E deficiency from MTRR (methionine synthase reductase) dysfunction (OMIM #236270) (summarized in Fig. 1). All these disorders manifest elevations in homocystine/homocysteine.

The methionine cycle allows for methionine to be converted to homocysteine which then recycles back to methionine (Fig. 1). Homocysteine can also be converted to cystathionine by Cystathionine beta synthase (CBS). Dysfunction of CBS leads to “classical homocystinuria” resulting in elevations of homocysteine and methionine. CBS is pyridoxine requiring and so some with CBS deficiency have a pyridoxine-responsive form.

The methionine cycle also requires MTHFR, MTR and MTRR to recycle homocysteine to methionine and these disorders have elevations in homocysteine, but low methionine (Fig. 1). MTR and MTRR require cobalamin as a cofactor and so elevated homocysteine can also be seen in the cobalamin processing defects (Fig. 1, yellow arrows).

CBS, MTHFR, MTR and MTRR are fairly rare and only limited information about oral health is available. Since poor oral health can increase risk for other health complications, then understanding whether the homocystinurias have a higher or similar risk to the general population is important. To that end, there are several things we do know about oral health in this population. Frequently dental crowding and high-arched palate are part of the description of CBS [1], but reports of other oral health complications are rarer. Limited reports of dental anomalies like hypoplastic teeth, white spot lesions and shortened roots have been seen in patients with metabolic homocystinurias [2,3]. More generally, oral disease is known to impact overall health [4].

Moreover, CBS deficiency is associated with osteopenia [1] which can impact oral health as one ages. The other homocystinurias from remethylation (and MTHFR) do not have oral health or bone density abnormalities reported [5,6], but this does not rule out impacts on oral health from these disorders because there is less knowledge about the non-CBS homocystinurias related to teeth issues. This review is attempting to determine if people with metabolic homocystinurias are more prone to dental issues like caries and periodontitis than would be expected.

## 2 Methods and cohort

Data were collected from patients with homocystinurias by a series of questionnaires using the RARE-X platform. RARE-X is a collaborative platform for global data sharing and a research program of Global Genes, a non-profit patient advocacy organization in rare diseases. This platform was designed to enable the collection of structured patient-reported data in affected domains for homocystinurias in conjunction with the patient advocacy group HCU America with the aim of providing a baseline natural history cohort study. It is designed for individuals to update their information longitudinally with reminders being sent on a regular basis, at minimum bi-annually. The current design is a series of questionnaires, that are triggered by the positive or negative answers from screening questions. Questionnaires are consistent across all disorders which have data collected by this research platform in terms of structure. All subjects were consented prior to the collection of their data. All research was performed in accordance with the Declaration of Helsinki. Demographic data were collected as the initial questionnaire and these data were derived from the oral health questionnaire following positive answers to oral health screening questions on the general health questionnaire and completed by the end of November 2022.

Due to the small size of the cohort, only descriptive statistics were done.

## 3 Results

Seventy-seven individuals with homocystinurias or their caregivers started the consenting process and enrolled, but only a total of 27 individuals/caregivers completed any of the questionnaires (15 caregivers and 12 patients) at the time of the data pull for this study. Thirteen individuals/caregivers provided answers to the oral health (48% of those who answered any questionnaire). Ten individuals had genetic testing done to confirm diagnoses, two are unsure and one did not have genetic testing. Three individuals had homocystinuria from a cobalamin disorder or MTHFR (further designation was not collected) whereas 10 were diagnosed with CBS deficiency (Table 1 summarizes the cohort who filled out the oral health questionnaire).

**Table 1:** Demographic information.

Diagnosis	Age (Years)	Person filling out questionnaire	Confirmation by genetic testing	Osteoporosis/osteopenia
CBS	10	CG	Yes	No
CBS	34	Patient	No	No
CBS	7	CG	Yes	No
CBS	51	Patient	Unsure	No
Not CBS	11	CG	Yes	No
CBS	38	Patient	Yes	Yes
Not CBS	15	CG	Yes	No
CBS	66	Patient	Yes	Yes
Not CBS	6	CG	Yes	No
CBS	40	Patient	Yes	Unsure
CBS	3	CG	Yes	No
CBS	46	Patient	Yes	No
CBS	46	Patient	Unsure	Yes

Gender assigned at birth is not included in Table 1 to maintain confidentiality. Of those who answered any one questionnaire, 70 % were assigned female at birth. The oral health response rate reflects this percentage. Sixty-one percent of respondents reported abnormalities ranging from scoliosis (32% if respondents) to osteoporosis (in 9% of respondents). Of this cohort 30.7% answered that they had osteoporosis/osteopenia or were unsure which is higher than the overall cohort.

Questionnaires covered a variety of topics including tooth structure, gum disease, and tooth alignment.

### 3.1 Tooth structure

Table 2 describes the tooth abnormalities in terms of structures and the percentages for each for this cohort.

**Table 2:** teeth structure.

	Abnormal baby teeth	Unusual number of teeth	Dental pulp abnormalities	Dental root abnormalities	Malformed teeth
Reported	1 (7%)	2 (15%)	1 (8%)	3 (23%)	2 (15%)
Not reported	12 (93%)	11 (85%)	11 (92%)	10 (77%)	11 (85%)
Unsure	0	0	0	0	0

Teeth begin their formation *in utero*, with primary teeth forming as early as 5 weeks *in utero* and permanent teeth hard tissue formation begins around birth. Disruptions in the formation stages can change the size, shape and number of teeth present. Abnormal baby teeth, unusual number of teeth and malformed teeth can present at this time.

Histopathological disruptions to the initiation and proliferation stages, prior to hard tissue formation, lead to anomalies in tooth number, which occur between 37 and 42 days *in utero*. Size and shape anomalies occur in the proliferation and morpho-differentiation stages as early as 55 days *in utero*. The development of teeth continues until children are into their teenage years with the development of their third molars [7].

Observations of malformation or quantity differences in terms of teeth implies some disturbance during *in utero* development and shortly after birth (when tooth formation is initiated). Interestingly all three of the individuals with Cobalamin disorders or MTHFR as a cause of their homocystinuria had either abnormal baby teeth or an unusual number of teeth (accounting for 7% and 15% of the whole cohort with these findings) see Table 2. The Cobalamin disorders and MTHFR can impact methylation and play a role in the creation of methyl groups from folate (Fig. 1).

Dental initiation of formation of permanent teeth begins between the ages of 4 months *in utero* and three years of age. Root changes seen in some studies in the maxillary incisors corresponds to tooth development between the ages of five and eleven years [7]. Because of the variety of timing for various dental anomalies, interruptions to teeth development in homocystinuria patients can occur at any time. Abnormal numbers of teeth do not predispose a child toward dental decay. However, other dental anomalies noted in some patients with homocystinurias such as white spot lesions or enamel hypoplasia [3] can predispose a person to dental decay [[8], [9], [10]] due to higher acid solubility and rougher surfaces encouraging cariogenic bacterial adhesion and colonization [11]. As a comparison to this cohort, hypomineralization or hypoplasia occurs in as many as 25% of the general population [7,12] (close to the percentage seen in this cohort of homocystinuria subjects).

### 3.2 Gum disease

Table 3 discussed the frequency of those who answered the oral health questionnaire that gum disease and related complications were seen.

**Table 3:** Gum disease.

	Fragile teeth	Gingivitis	Gum Disease
Reported	1 (7%)	4 (31%)	2 (15%)
Not reported	11(86%)	9 (69%)	11(85%)
Unsure	1 (7%)	0	0

The dental definition of gingivitis is a localized gingival inflammation [13]. Gingivitis can be affected by numerous factors such as genetics, medical history, hormones, oral hygiene and diet [13]. Here, a number of individuals report gingivitis (31%) (Table 3), however, gingivitis occurs in half of the population by age four and peaks around 100% in puberty [13], thus the results here are not outside the expected.

On the other hand, periodontitis is inflammation of the supporting structures, including the bone, and may be a better measure of dental disease [13]. The question in the questionnaire is about the presence of gum disease with 15% having this concern.

### 3.3 Jaw and teeth arrangements

Table 4 explores the findings in this cohort concerning the structure of teeth. Alignment of dentition is how the teeth are arranged with regards to rotations and displacement. Occlusion is how the jaws and teeth fit together when someone is biting down. Malalignment and malocclusion are disturbances in either of these. Malalignment is often related to the direction the forming tooth buds are facing as the tooth is developing, as well as can be influenced by spacing issues impacting how the jaws fit together. Malocclusion can be divided further into two subsets: dental malocclusion and skeletal malocclusion. Dental malocclusion is how the teeth fit together. Skeletal malocclusion is how the jawbones fit together.

**Table 4:** Structure.

	Mal-occluded teeth	Bruxism	Mal-aligned
Reported	10 (77%)	4 (31%)	5 (38%)
Not reported	3 (23%)	9 (69%)	7 (55%)
Unsure	0	0	1 (7%)

Malalignment is seen in not just those with CBS but also all three of the other homocystinurias patients. Mal-occluded teeth are only being seen in the CBS deficiency patients. Malocclusion and malalignment are both related to the positioning of teeth in the jaw bones and a high arched palate or other bony malformations can be explained by these positioning issues. For example, when teeth are crowded together thereby decreasing the dental arch length, narrow and arched palates are common as the tongue has less transverse space to sit within. The tongue then rests higher in the oral cavity leading to a higher or arched palate. With discrepancies in the transverse relationship of the maxilla as seen in high arched palates, it is possible to have discrepancies in both the dental relationship and the skeletal relationship of the maxilla and mandible. Changes in the bony formation of the mandible, with specific regards to the condyle, which can often be seen in patients with osteopenia and osteoporosis, can lead to additional jaw discrepancies and alignment as the condyl of the mandible no longer rests appropriately in the condylar fossa of the skull.

## 4 Discussion

The current guidelines for diagnosis and management of CBS deficiency, remethylation disorders and cobalamins [1,5] do not address oral health concerns except for mentioning that individuals with CBS deficiency can have facial structure differences (thus resulting in maloccluded teeth and narrow palates). Here we have a small number of individuals with homocystinurias who answered this questionnaire about oral health. We have reported some tooth structure differences, similar rates of gingivitis, and potentially increased malalignment of teeth compared to the general United States population.

In our small cohort of MTHFR and cobalamin disorder homocystinurias, several individuals had abnormalities related to “baby” teeth number and structure. This was not observed in those with CBS deficiency. Since teeth formation starts *in utero,* one must question whether the patient's underlying disorder impacting folate or methylation could be explained prenatally. Moreover maternal folate consumption has an important role in tooth development as observed by Dhamo [14], but that role is still not fully understood. *Via* their prospective cohort study, they were able to observe that folate supplementation pre-conceptionally or post-conceptionally can affect the dental age (speed of development of dentition) by 1–2 months in children. However, *concentrations* of the folate, as well as Vitamin B12, did not matter with regards to accelerated or decelerated child dental age. Further, maternal MTHFR polymorphism did not have a measurable effect on the relationship between folate and Vitamin B12 consumption and the child's dental developmental velocity (Dhamo). It has been theorized that folate may stimulate certain inhibitors with regards to tooth mineralization (Dhamo), specifically an inhibitor called pyrophosphate [15], which can explain some of the hard tissue abnormalities seen in this population that supplements with folate.

Although the non-CBS deficiency homocystinuria patients had more abnormal teeth, in general our homocystinuria population reports better gingival health than the general United States population [13]. To that end, folate and Vitamin B12 have been shown to be protective for gingival health, as has been seen in other studies [16]. For example, in pediatric patients with gingival overgrowth due to medications, folate supplementation has helped mitigate the magnitude of medication induced gingival overgrowth [17] and in adult populations, folic acid supplementation showed a positive correlation with a decrease on bleeding on probing, the gold standard for diagnosing gingival disease [18]. As part of therapy, individuals with homocystinurias frequently supplement folic acid, folate or follinic acid and perhaps this is the reason the numbers are quite low for gingivitis in this population. However, folate and its derivatives preventing gingivitis may not be the only possible explanation for this observation. Other hypotheses include having one of the homocystinurias is actually protective against gingivitis and other periodontal diseases, or that there is an under-reporting of gingival disease by those who answered this questionnaire.

Understanding gum disease is important in this cohort since gingivitis is a reversible disease and is a necessary prerequisite for the development of periodontitis, progressive connective tissue destruction, and bone loss [13]. Given the risk especially with CBS deficiency of osteopenia and osteoporosis, monitoring and controlling for gingivitis may play an important role in maintenance of oral health as this patient population ages.

Perhaps a better measure for this population would be measuring the level of periodontitis since it is an indication of bony changes and, thus, may be more useful for examining whether the risk to bone in homocystinurias can result in change in the oral cavity. Much has been documented on the impact of cobalamin deficiency has on odontogenic development, but little has been documented about periodontal disease development. Periodontal disease can have devastating effects to the longevity of the teeth due to bony changes of the support structure of teeth. In this cohort, both of the patients with gum disease are older (>40 years) and have CBS deficiency and so the question exists as to whether this gum disease is in fact a manifestation of the osteopenia. Patients with CBS deficiency predominately have skeletal issues including inappropriate growth patterns (if inadequately treated) [1] and high palates (potentially related to growth). Bone formation with regards to microcephaly and palate malformation has been seen in mice with cobalamin deficiency [19]. In terms of oral health, both malalignment and malocclusion could be related to skeletal issues because teeth form in dental tooth buds and their subsequent trajectory is affected by quality of bone. Skeletal mass is associated with malocclusion, showing there may be a relationship between osteopenia and malocclusion [20]. Here we see malocclusion in our CBS patients predominately and malalignment in both.

Dental root abnormalities are described in Björksved for CBS deficiency [2]. All of our cohort who reports of dental root abnormalities have CBS deficiency [2]. In the Björksved study, maxillary incisors of all 14 patients who followed up for clinical exam were short compared to reference values [2]. Our cohort does not see quite this level of reported root problems, but given that all with this complication have CBS deficiency, we cannot rule out the impact of osteoporosis/osteopenia.

Hypoplastic or hypomineralization occurs in as much as 25% of the population on the permanent molars [7]. The data set shows one person who has fragile teeth, two people with malformed teeth and 1 person with abnormal baby teeth. These could all be different ways to describe hypomineralized or hypoplastic teeth. Other studies have showed hypoplastic teeth in patients with homocystinuria [3]. This study shows that dental abnormalities related to enamel quality such as hypoplastic or hypomineralized teeth are in line with expectations in the general population without homocystinurias. Other studies have shown that similar diseases can have similar findings of hypoplastic teeth. Bassim showed some patients with methylmalonic acidemia had severely hypoplastic teeth, while most patients with methylmalonic acidemia have normal teeth. He concluded there is likely a relationship between methylmalonic acidemia and enamel developmental pathology to a certain extent, possible related to specific genotypes [21].Dental avoidance and fear are common in the United States population, with some studies showing >29% of the general population having high or moderate dental fear [22]. This same study showed 36.5% of respondents had not been to the dentist in the last year [22]. A complicating factor in treating patients with homocystinuria and CBS deficiencies is that use of nitrous oxide as an anxiolysis medication is contraindicated due to inactivation of cobalamin and subsequent increase in homocysteine concentrations in the blood [1]. While in line with the general population with regards to incidence of hypoplastic and hypomineralized teeth, avoidance to dental care in homocystinuria population can be especially detrimental as they have fewer anxiolysis options for dental treatment. It is unclear whether dental avoidance played a role in the numbers of individuals who completed this survey or whether those who did not complete the survey but completed other surveys did not have oral health complications. This is one of the limitations of a questionnaire-based survey.

As summary, individuals with the homocystinurias have a number of oral challenges, but many of these challenges are in line with the general population. Some of these are likely related to bone differences especially seen in CBS deficiency (*e.g.*, malalignment, malocclusion) which are better characterized in the literature. Other differences such as a potential *in utero* effect (especially seen in Cobalamin disorders and severe MTHFR) have not been well characterized. Given the small size of this cohort, additional studies are necessary, but oral health has been especially important in this cohort, and we recommend on-going dental interventions including regular cleanings to try to stave off gum disease (*i.e.*, periodontal disease) for which these patients may have a greater risk. Any increase in caries in this population are likely more a result of oral hygiene and diet, and less due to homocystinurias inherently, given that the percentages of defects seen in this population are on par with the general population.

## Declaration of Competing Interest

None.

## Data Availability

Data will be made available on request.
